# Mediterranean diet improves blastocyst formation in women previously infected COVID-19: a prospective cohort study

**DOI:** 10.3389/fnut.2024.1371077

**Published:** 2024-06-20

**Authors:** Huijun Chen, Jing Wang, Hongxin Guo, Qi Zhao, Ge Lin, Berthold Hocher, Philipp Kalk, Zetao Wang, Fei Gong

**Affiliations:** ^1^Clinical Research Center for Reproduction and Genetics in Hunan Province, Reproductive and Genetic Hospital of CITIC-Xiangya, Changsha, Hunan, China; ^2^Xiangyang Central Hospital, Affiliated Hospital of Hubei University of Arts and Science, Xiangyang, Hubei, China; ^3^Department of Nephrology, Charité Universitätsmedizin Berlin, Berlin, Germany; ^4^Institute of Reproductive and Stem Cell Engineering, NHC Key Laboratory of Human Stem Cell and Reproductive Engineering, School of Basic Medical Science, Central South University, Changsha, Hunan, China; ^5^Key Laboratory of Stem Cells and Reproductive Engineering, Ministry of Health, Changsha, China; ^6^Fifth Department of Medicine (Nephrology/Endocrinology/Rheumatology/Pneumology), University Medical Centre Mannheim, University of Heidelberg, Mannheim, Germany; ^7^Institute of Medical Diagnostics, IMD, Berlin, Germany; ^8^Diaverum Renal Care Center, Diaverum MVZ Am Neuen Garten Standort Ludwigsfelde, Potsdam, Germany; ^9^Department of Gastroenterology, The First Affiliated Hospital of Harbin Medical University, Harbin, Heilongjiang, China

**Keywords:** mediterranean diet, post COVID-19, IVF outcomes, embryo, pregnancy

## Abstract

**Objectives:**

Our study tries to investigate the effect of the Mediterranean diet (MeDiet) on assisted reproductive treatment outcomes in women after COVID-19 infection.

**Design:**

A prospective observational cohort study in the Reproductive and Genetic Hospital of CITIC-Xiangya from February 2023 to August 2023.

Subjects: A total of 605 participants previously infected with COVID-19 were enrolled.

**Exposure:**

None.

**Main outcome measurement:**

The primary outcomes are oocyte and embryo quality. The secondary outcomes are pregnancy outcomes.

**Results:**

A majority of participants (*n* = 517) followed low to moderate MeDiet, and only a small group of them (*n* = 88) followed high MeDiet. The blastocyst formation rate is significantly higher in MeDiet scored 8–14 points women (46.08%), compared to the other two groups (which is 41.75% in the low adherence population and 40.07% in the moderate adherence population respectively) (*p* = 0.044). However, the follicle number on hCG day, yield oocytes, normal fertilized zygotes, fertilization rate, day three embryos (cleavage embryos), and embryo quality are comparable among the three groups. For those who received embryo transfer, we noticed an obvious trend that with the higher MeDiet score, the higher clinical pregnancy rate (62.37% vs. 76.09% vs. 81.25%, *p* = 0.197), implantation rate (55.84% vs. 66.44% vs. 69.23%, *p* = 0.240) and ongoing pregnancy rate (61.22% vs. 75.00% vs. 81.25%, *p* = 0.152) even though the *p* values are not significant. An enlarging sample size study, especially in a high adherence population should be designed to further verify the effects of MeDiet’s role in improving IVF performance.

**Conclusion:**

High adherence to MeDiet is associated with improved blastocyst formation in women after COVID-19 infection. There is also a trend that high adherence to MeDiet might be beneficial to clinical pregnancy, embryo implantation as well as ongoing pregnancy in these women.

## 1 Introduction

The Mediterranean diet (MeDiet) is a dietary pattern inspired by the traditional eating habits of countries bordering the Mediterranean Sea, such as Greece, Italy, and Spain (1). This dietary pattern was first coined by Ancel Keys back in 1960 (2). It is characterized by an abundance of fruits, vegetables, whole grains, legumes, and nuts, with olive oil as a primary source of fat. Fish and poultry are consumed in moderate amounts, while red meat is limited (1, 3, 4). The diet is known for its potential health benefits, including reducing the risk of cardiovascular disease (5, 6), diabetes (7, 8), cancers (9, 10), and overall mortality (11), promoting weight management and lowering the risk of metabolic syndrome (12, 13), and providing essential nutrients.

The Mediterranean diet is also said to offer a promising and relatively straightforward approach to mitigating the severity of COVID-19 infection (14). R. Perez-Araluce et al. revealed that individuals demonstrating moderate to high adherence to the Mediterranean diet experienced a significantly reduced likelihood of contracting COVID-19 (15). Notably, observational studies have emphasized a correlation between adherence to the Mediterranean diet and improved outcomes in individuals with COVID-19 (such as mortality and recovery rate), as well as a reduced risk of COVID-19 infection across various populations (16, 17). It is also recommended as a useful nutritional approach for patients with post-COVID-19 syndrome (18).

Moreover, MeDiet is associated with the improvement of female infertility, decreasing the risk of developing pregnancy-associated complications (19). Published evidence also revealed MeDiet’s role in assisted reproduction. A previous cohort study investigated the Mediterranean diet’s effect on *in vitro* fertilization (IVF) outcomes and it turned out that the higher MeDiet adherence group showed more embryos available (8.40 ± 5.26 vs. 7.40 ± 4.71, *p* = 0.028) while the pregnancy rate and implantation rate was similar (20). However, another study showed that women with higher Mediet scores had significantly higher clinical pregnancy rates (50.0% vs. 29.1%, *p* = 0.01) and live birth rates (48.8% vs. 26.6%, *p* = 0.01) (21). Conversely, an Italian study finds that the Mediterranean diet score was not significantly associated with IVF outcomes (22). A recent meta-analysis including 11 studies also concludes that insufficient current evidence exists to support the clinical application of high adherence to the Mediterranean diet and fertility markers (23). More evidence of well-designed clinical studies is needed to prove the comprehensive role of Mediet in IVF outcomes.

Current evidence demonstrates that COVID-19 infection impairs reproductive function and leads to infertility as well as unsuccess in IVF treatment (24). In a small-sample observational study, a reduction in the proportion of top-quality embryos was observed in women post-COVID-19 infection (25). Additionally, a slight decrease in the blastocyst (day 5 or later embryo) formation rate was recorded in the case group (26). Based on these findings MeDiet is helpful in many pathological situations of COVID-19 infection and is still controversial in IVF outcomes. Thus, our study tries to investigate the effect of MeDiet on IVF outcomes in women after COVID-19 infection.

## 2 Materials and methods

### 2.1 Study design and setting

We performed a prospective observational cohort study in women undergoing assisted reproductive technology (ART) treatment from February 2023 to August 2023.

### 2.2 Ethical approval

The current study was approved by the Ethics Committee of the Reproductive and Genetic Hospital of CITIC-Xiangya (approval number: LL-SC-2023-012) and written consent was obtained from all participating patients.

### 2.3 Participants

Women who were infected with COVID-19 before IVF treatment would be eligible for enrollment. Inclusion criteria: (1) age between 18 and 45 years and willingness to participate in the study, (2) women received ovarian stimulation, (3) the maximal time from COVID-19 infection to IVF treatment was defined as half a year, (4) only the first cycle following COVID-19 recovery was included. Exclusion criteria were as follows: (1) oocyte donation, (2) intrauterine insemination, (3) oocyte cryopreservation, (4) never being affected with COVID-19, (5) ART contraindications according to the guideline, such as either the man or the woman suffering from severe mental disorders, acute infections of the genitourinary system, or sexually transmitted diseases.

### 2.4 Sample size calculation

In this study, we considered a clinical pregnancy rate difference of 5% to detect a statistically significant difference, with a test power of 90% and a set α of 0.05. The calculated sample size required for the study was 594 participants. Accounting for a dropout rate of 5%, the final sample size for the study is 624.

### 2.5 Questionnaire and MeDiet score

The questionnaire, which consists of 14 questions, was published in a lot of journals and widely used to evaluate adherence to the Mediterranean diet (27). The questionnaire focused on the category and consumption of food and drinks in daily life such as olive oil, fresh vegetables and fruits, seafood, and grains and nuts. Each question can be scored 1 point, with a total of 14 points. A higher score indicates higher adherence to the Mediterranean diet (Table 1).

**Table 1:** Mediterranean diet questionnaire and answer distribution.

Questions	Score		Frequency	
	1	0	1	0
1.Do you use olive oil as the principal source of fat for cooking?	Yes	No	27	578
2.How much olive oil do you consume per day, including that used in frying, salads, meals eaten away from home, etc.?	≥54 g	<54 g	74	531
3.How many servings of vegetables do you consume per day? (count garnish and side servings as ½ point; a full serving is 200 g)	≥2	<2	362	243
4.How many servings of fruit do you eat per day (including fresh fruit juice)?	≥3	<2	123	482
5.How many servings of red meat/burgers/sausages do you consume per day (a full serving is 100-150 g)?	<1	≥1	309	296
6.How many servings of (artificial) cream/butter/cheese do you consume per day (a full serving is 12 g)?	<1	≥1	551	54
7.How many carbonated and/or sugar-sweetened beverages do you consume per day?	<1	≥1	535	70
8.What is your weekly alcohol consumption?	≥700 mL	<700 mL	48	557
9.How many servings of legumes do you consume per week (a full serving is 150 g)?	≥3	<3	179	426
10.How many servings of fish/seafood do you consume per week? (100-150 g of fish, 4-5pieces or 200 g of seafood)	≥3	<3	128	477
11.How often do you consume (non-homemade) pastries/cookies/cakes per week?	<2	≥2	462	143
12.How many servings of nuts do you eat per week (a full serving is 30 g)?	≥3	<3	126	479
13.Do you prefer chicken/turkey/rabbit over beef/pork/burgers/sausages?	Yes	No	159	446
14.How often do you consume boiled vegetables/pasta/rice or tomatoes/garlic/onions/chives sautéed in olive oil per week?	≥2	<2	240	365

Infertile couples were informed about the importance of lifestyle and dietary habits for preparing for pregnancy on their first visit to the hospital. Advise was given to them to adopt a healthy lifestyle and dietary habits for at least 1 month. Which includes a high intake of olive oil, fruit, nuts, vegetables, and cereals; a moderate intake of fish and poultry; a low intake of dairy products, red meat, processed meats, and sweets; and wine in moderation, consumed with meals (28). Most commonly, the recommended numbers of servings for these food groups are represented as a diet pyramid. A diet pyramid is considered a useful way to display the general principles of a diet including approximate recommendations for quantities of food groups (i.e., those consumed in the greatest quantities appear in the largest section of the pyramid) (29). Study participants were required to complete the Mediterranean diet questionnaire at the onset of ovulation stimulation treatment, providing information based on their actual dietary habits. The data was collected afterward and analyzed.

### 2.6 Outcome measurement

The clinical pregnancy was identified as the presence of gestational sac(s) exhibiting fetal heart activity through ultrasound in the fourth week following embryo transfer. The implantation rate was calculated by dividing the total number of embryos transferred by the number of sacs. Subsequently, miscarriage was characterized as the loss of intrauterine pregnancy after the confirmation of gestational sacs by ultrasound (30).

### 2.7 Data analysis

In the data processing procedure, all the missing data will be excluded from the final analysis. Data analyses were conducted using Statistical Package for Social Sciences for Windows, version 25.0 (SPSS Inc., Chicago, IL, United States). The homogeneity of variance and normality of data were assessed using the Levene and Kolmogorov–Smirnov tests, respectively. Results were presented as medians (interquartile ranges), means ± standard deviation, or frequency (%). Group comparisons for quantitative variables employed the Kruskal-Wallis test or ANOVA based on normality, while qualitative variables were compared using the Chi-square test or Fisher’s exact test. Statistical significance was defined as a two-sided *p*-value <0.05.

## 3 Results

**Figure 1:**
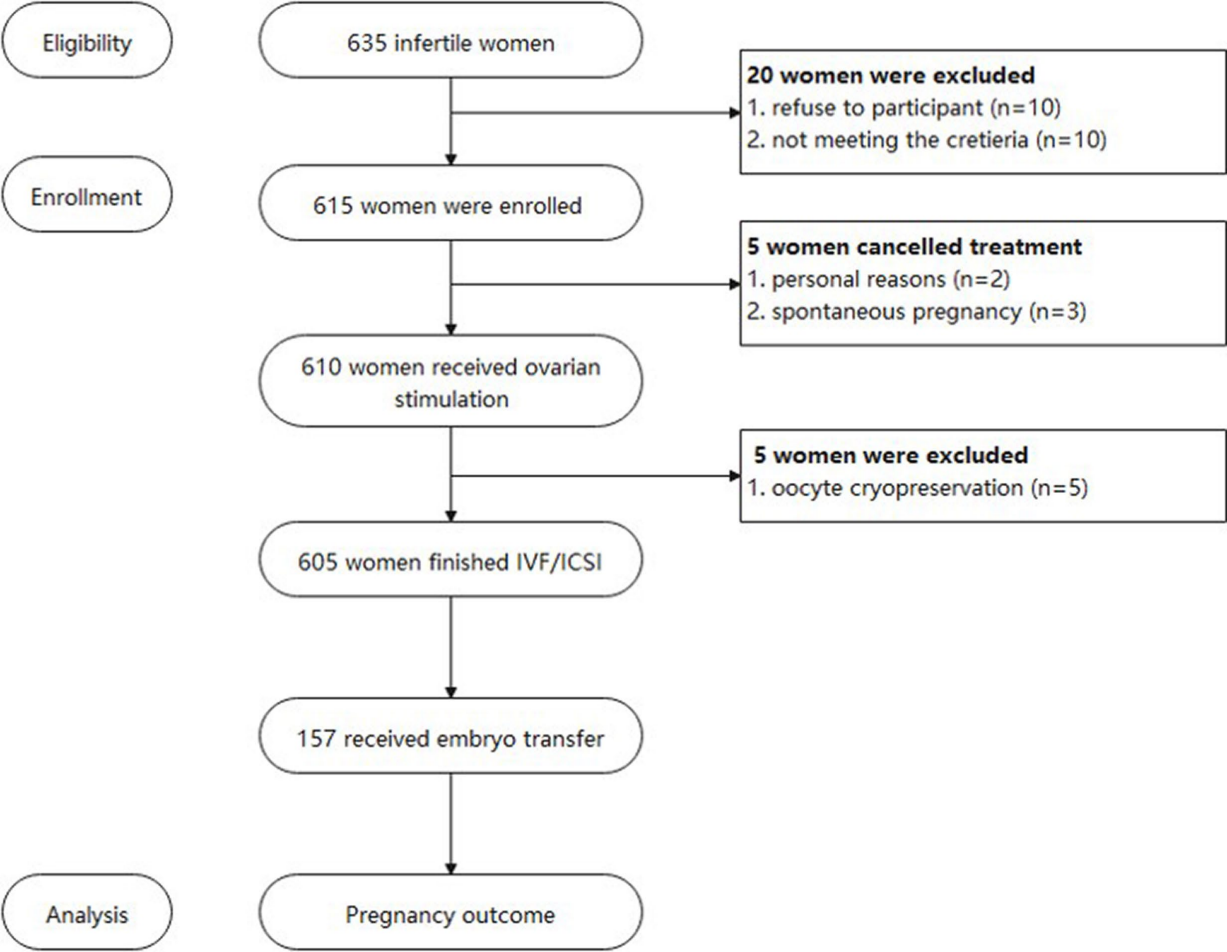
Flow chart of the study.

**Figure 2:**
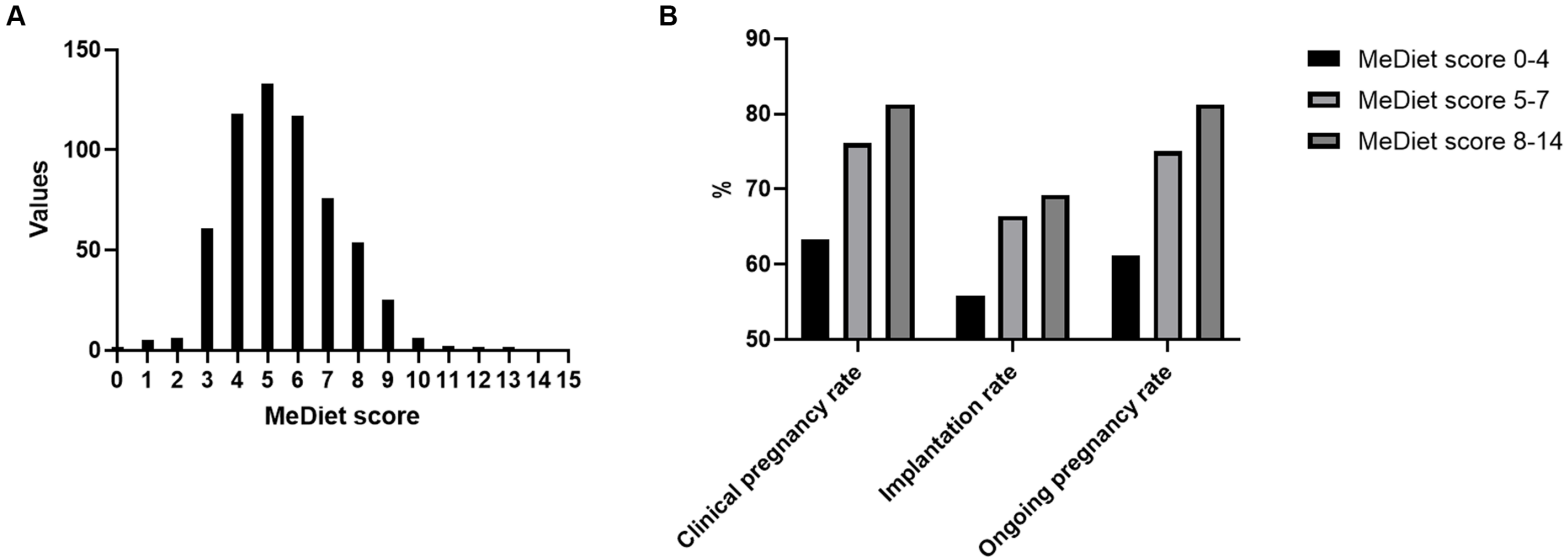
MeDiet score distribution and pregnancy outcomes.

A total of 635 participants were recruited for the study, while 10 of them refused to fulfill the questionnaire and 10 of them did not meet the inclusive criteria, and the remaining 615 were enrolled in our study. However, 5 of them accepted oocyte cryopreserve due to personal reasons. 3 participants conceived spontaneously and 2 participants did not start the IVF treatment at the end of the study, leaving 605 participants to finish the ART procedure and their data was analyzed (Figure 1). Table 1 shows the 14 questions of the questionnaire and the answer distribution. The MeDiet score distribution is shown in Figure 2A. We divided our participants into three groups according to the MeDiet score: low adherence [MeDiet score 0–4 (*n* = 191)], medium adherence [MeDiet score 5–7 (*n* = 326)] and high adherence [MeDiet score 8–14 (*n* = 88)]. A majority of participants followed low to moderate MeDiet, and only a small group of them followed high MeDiet. There is no difference in demographic information such as age, infertility type and reason, body mass index, waist-to-hip ratio, blood pressure, etc. among these groups. Besides, we also evaluated some serological markers to evaluate the metabolic status of our participants. No difference was found in fasting glucose, insulin level, HOMA-IR (homeostatic model assessment of insulin resistance) as well as thyroid function among groups. Similarly, no differences were found in blood cells and coagulation indicators in our participants (Table 2).

**Table 2:** The demographic and baseline information of participants.

	MeDiet score 0–4 (*n* = 191)	MeDiet score 5–7 (*n* = 326)	MeDiet score 8–14 (*n* = 88)	*p* value
Age (year)	32.00 (29.00, 36.00)	32.00 (29.00, 35.25)	33.00 (31.00, 36.75)	0.104
Infertility type (%)				
Primary	36.65 (70/191)	30.67 (100/326)	35.23 (31/88)	0.570
Secondary	55.50 (106/191)	58.28 (190/326)	54.55 (48/88)	
Others	7.85 (15/191)	11.04 (36/326)	10.23 (9/88)	
Infertility reason (%)*				
PCOS	19.37 (37/191)	19.94 (65/326)	2045 (18/88)	0.976
Endometriosis	32.98 (63/191)	38.96 (127/326)	29.55 (26/88)	0.168
Oviduct malfunction	64.92 (124/191)	66.26 (216/326)	63.64 (56/88)	0.884
Infertility duration (year)	3.00 (1.00, 4.00)	3.00 (1.00, 4.00)	3.00 (1.25, 5.00)	0.382
BMI (kg/m2)	22.43 (20.24, 24.03)	22.37 (20.28, 24.22)	21.57 (20.34, 23.83)	0.713
Waist-to-hip ratio	0.81 (0.77, 0.85)	0.80 (0.77, 0.85)	0.81 (0.77, 0.85)	0.932
AMH (ng/ml)	3.14 (1.57, 4.91)	2.79 (1.60, 4.84)	2.66 (1.45, 4.47)	0.410
AFC	21.00 (10.00, 33.00)	22.00 (13.00, 33.00)	18.50 (9.00, 30.00)	0.228
Basal FSH (mIU/ml)	6.37 (5.32, 7.88)	6.24 (5.14, 7.83)	6.55 (5.40, 8.59)	0.275
Basal LH (mIU/ml)	4.44 (2.77, 6.77)	4.25 (2.66, 6.22)	4.23 (2.66, 6.47)	0.642
Basal E2 (pg/ml)	38.60 (29.78, 97.00)	42.05 (30.98, 91.35)	40.10 (29.60, 130.00)	0.571
Basal P (ng/ml)	0.32 (0.18, 1.08)	0.38 (0.19, 1.23)	0.39 (0.22, 1.57)	0.441
Fasting glucose (mmol/L)	5.15 (4.91, 5.44)	5.20 (4.97, 5.42)	5.15 (4.86, 5.44)	0.534
Fasting insulin (μIU/mL)	8.00 (5.70, 10.60)	7.60 (5.40, 10.91)	7.10 (5.03, 9.90)	0.299
HOMA-IR	1.69 (1.19, 2.43)	1.75 (1.23, 2.43)	1.62 (1.03, 2.40)	0.294
Free T3 (pg/ml)	2.86 (2.64, 3.09)	2.82 (2.60, 3.07)	2.73 (2.50, 3.03)	0.073
Free T4 (ng/ml)	1.00 (0.92, 1.09)	0.99 (0.92, 1.09)	0.99 (0.89, 1.07)	0.416
TSH (μIU/ml)	1.90 (1.37, 2.57)	1.76 (1.23, 2.55)	1.61 (1.13, 2.37)	0.100
Systolic blood pressure (mmHg)	114.00 (106.00, 122.00)	115.00 (105.00, 122.00)	111.00 (102.00, 121.00)	0.184
Diastolic blood pressure (mmHg)	73.00 (67.00, 81.00)	74.00 (68.00, 80.00)	72.00 (65.00, 79.00)	0.524
WBC (x10^9/L)	5.90 (5.10, 7.40)	5.90 (5.01, 7.20)	6.20 (5.00, 7.38)	0.781
RBC (x10^12/L)	4.49 (4.22, 4.71)	4.43 (4.23, 4.66)	4.39 (4.21, 4.69)	0.347
HGB (g/L)	135.00 (128.00, 140.00)	133.00 (127.00, 139.00)	135.00 (127.25, 139.00)	0.403
MCV (fl)	90.10 (87.70, 92.80)	90.60 (87.68, 93.20)	90.80 (88.33, 93.28)	0.568
PLT (x10^9/L)	240.00 (201.00, 282.00)	239.00 (202.00, 281.50)	245.00 (212.00, 294.50)	0.713
D-Dimer (mg/L)	0.23 (0.16, 0.40)	0.24 (0.15, 0.35)	0.25 (0.15, 0.32)	0.873
APTT (s)	33.40 (31.50, 35.60)	33.05 (31.00, 35.20)	32.30 (30.20, 35.28)	0.104
PT (s)	11.00 (10.70, 11.40)	11.10 (10.70, 11.50)	11.00 (10.50, 11.50)	0.336
FIB (g/L)	2.76 (2.50, 3.15)	2.82 (2.47, 3.11)	2.90 (2.62, 3.24)	0.194
TT (s)	14.00 (13.30, 14.80)	14.00 (13.20, 14.90)	13.90 (13.20, 14.90)	0.949

PCOS, polycystic ovary syndrome; BMI, body mass index; AMH, anti-Müllerian hormone; AFC, antral follicle count; FSH, follicle-stimulating hormone; LH, luteinizing hormone; E2: estradiol; P, progesterone; HOMA-IR, homeostatic model assessment of insulin resistance; T3, triiodothyronine; T4, thyroxine; TSH, thyroid-stimulating hormone; WBC, white blood cell count; RBC, red blood cell count; HGB, hemoglobin; MCV, mean corpuscular volume; PLT, platelet; APTT, activated partial thromboplastin time; PT, prothrombin time; FIB, fibrinogen; TT, thrombin time. *Some participants have more than one infertility reason.

Ovarian stimulation protocol differs among the groups with less gonadotropin-releasing hormone agonist (GnRH-a) protocol while more uncommon protocols (such as mild stimulation, letrozole protocol, etc.) are administrated in the higher MeDiet score group (*p* = 0.019). Nevertheless, there is no difference in sex hormones such as estradiol (E2), progesterone (P), and luteinizing hormone (LH) on human chorionic gonadotropin (hCG) day. The follicle number on hCG day, yield oocytes (including metaphase II (MII), metaphase I (MI), germinal vesicle (GV), degenerated oocytes), 2 pronucleus (2PN) zygotes, fertilization rate, day three embryos (cleavage embryos) and embryo quality are comparable among the three groups. Interestingly, there is an obvious difference in that the blastocyst formation rate is significantly higher in MeDiet scored 8–14 points women (46.08%), compared to the other two groups (which is 41.75 and 40.07% respectively) (*p* = 0.044). Further multivariate regression analysis also shows the positive relationship between MeDiet and blastocyst formation [adjusted β:0.077, 95% confidential interval: (0.028, 0.313), *p* = 0.019]. Moreover, age is negatively related to blastocyst formation [adjusted β: −0.099, 95% confidential interval: (−0.098, −0.018), *p* = 0.005] (Supplementary Table S1). However, there is no difference in blastocyst quality (Table 3).

**Table 3:** Ovarian stimulation outcomes.

	MeDiet score 0–4 (*n* = 191)	MeDiet score 5–7 (*n* = 326)	MeDiet score 8–14 (*n* = 88)	*p* value
**Protocol (%)**				
GnRH-agonist protocol	37.70 (72/191)	41.41 (135/326)	27.27 (24/88)	**0.019**
GnRH-antagonist protocol	37.17 (71/191)	41.72 (136/326)	39.77 (35/88)	
PPOS	17.28 (33/191)	11.35 (37/326)	19.32 (17/88)	
Others*	7.85 (15/191)	5.52 (18/326)	13.64 (12/88)	
Gn dosage/[IU]	2250.00 (1537.50, 2975.00)	2306.25 (1612.50, 3000.00)	2193.75 (1650.00, 2971.88)	0.806
Gn duration/[day]	10.00 (9.00, 12.00)	10.00 (9.00, 12.00)	10.00 (9.00, 11.25)	0.428
E2 on hCG day/[pg/ml]	2931.50 (1819.00, 4437.75)	2983.50 (1577.75, 4285.00)	2540.00 (1531.00, 3874.00)	0.271
P on hCG day/[ng/ml]	0.72 (0.49, 1.11)	0.71 (0.47, 1.04)	0.67 (0.46, 1.03)	0.451
LH on hCG day/[mIU/ml]	2.23 (1.53, 3.67)	2.30 (1.58, 3.79)	2.59 (1.51, 4.54)	0.423
hCG dosage for triggering (IU)	5000.00 (5000.00, 6000.00)	5000.00 (2000.00, 6000.00)	5000.00 (2000.00, 6000.00)	0.226
Follicles on hCG day	11.00 (7.00, 14.00)	11.00 (7.00, 14.00)	10.00 (5.00, 13.00)	0.226
No. of oocytes retrieved	11.00 (6.00, 14.00)	10.50 (6.00, 14.00)	10.00 (4.25, 12.75)	0.149
MII oocytes	9.00 (5.00, 12.00)	9.00 (5.00, 13.00)	8.00 (3.25, 11.75)	0.161
MI oocytes	0.72 ± 1.59	0.79 ± 1.28	0.53 ± 1.13	0.092
GV oocytes	0.85 ± 1.54	0.69 ± 1.24	0.76 ± 1.70	0.701
Degenerated oocytes	0.14 ± 0.36	0.17 ± 0.41	0.16 ± 0.37	0.831
No. of 2PN zygotes	6.00 (3.00, 9.00)	6.00 (3.00, 9.00)	6.00 (3.00, 8.00)	0.259
Fertilization methods (%)				
IVF	49.74 (95/191)	46.63 (152/326)	45.45 (40/88)	0.942
ICSI	43.98 (84/191)	46.93 (153/326)	48.86 (43/88)	
IVF + ICSI	6.28 (12/191)	6.44 (21/326)	5.68 (5/88)	
Fertilization rate (%)	61.54 (50.00, 76.44)	62.50 (50.00, 76.92)	66.67 (42.86, 82.58)	0.560
Cleavage embryos	8.00 (5.00, 11.00)	7.00 (4.00, 11.00)	6.50 (3.00, 10.75)	0.268
The number of day 3 good quality embryo	4.00 (1.00, 6.00)	3.00 (1.00, 5.00)	2.50 (1.00, 5.00)	0.413
Blastocyst formation rate (%)	41.75 (516/1236)	40.07 (864/2156)	46.08 (235/510)	**0.044**
The number of good-quality blastocysts	0.29 ± 0.88	0.32 ± 0.89	0.31 ± 0.98	0.820

GnRH, gonadotropin-releasing hormone; PPOS, progestin-primed ovarian stimulation; Gn, gonadotropin; LH, luteinizing hormone; E2, estradiol; P, progesterone; hCG, human chorionic gonadotropin; MII, metaphase II (reflects oocytes quality, only MII oocytes can be fertilized); MI, metaphase I; GV, germinal vesicle; 2PN, pronucleus; IVF, in vitro fertilization; ICSI, intracytoplasmic sperm injection; Good quality embryo is defined as D3 embryo ≥ 7C-II and blastocyst ≥ 4BB while fair embryo is defined as D3 embryo <7C-II and blastocyst <4BB; Data are given as medians (interquartile ranges), means ± standard deviation, or number (percentage). *Others include mild stimulation, Gn stimulation, letrozole protocol, and natural cycle.

Bold values present *p*<0.05, means significantly different.

There were only 157 women out of 605 participants received fresh embryo transfer. The reasons for other participants’ embryo transfer cancellation are as follows: 1. Ovarian hyperstimulation syndrome (*n* = 15), 2. No oocytes retrieved (*n* = 5), 3. No transferrable embryo (*n* = 35), 4. Desynchronization between the endometrium and the embryo (*n* = 165), 5. Peri-implantation genetic test (*n* = 169), 6. Personal reasons (*n* = 59). The follow-up in this study ends 3 months after the embryo transfer. For those who received embryo transfer, we noticed an obvious trend that with the higher MeDiet score, the higher clinical pregnancy rate (62.37% vs. 76.09% vs. 81.25%, *p* = 0.197), implantation rate (55.84% vs. 66.44% vs. 69.23%, *p* = 0.240) and ongoing pregnancy rate (61.22% vs. 75.00% vs. 81.25%, *p* = 0.152) even though the *p* values are not significant (Table 4; Figure 2B). Only two miscarriage cases and no ectopic pregnancy cases were observed in the study population.

**Table 4:** Pregnancy outcomes.

	MeDiet score 0–4 (*n* = 49)	MeDiet score 5–7 (*n* = 92)	MeDiet score 8–14 (*n* = 16)	*p* value
Endometrium thickness (mm)	12.50 (11.05, 14.00)	12.40 (11.00, 13.80)	13.20 (11.80, 14.55)	0.306
The number of embryos transferred	2.00 (1.00, 2.00)	2.00 (1.00, 2.00)	2.00 (1.00, 2.00)	0.931
Good-quality embryo transferred	1.00 (0.00, 2.00)	1.00 (0.00, 2.00)	1.50 (0.00, 2.00)	0.988
Clinical pregnancy rate (%)	63.27 (31/49)	76.09 (70/92)	81.25 (13/16)	0.197
Implantation rate (%)	55.84 (43/77)	66.44 (97/146)	69.23 (18/26)	0.240
Early miscarriage rate (%)	2.04 (1/49)	1.09 (1/92)	0	0.729
Ectopic pregnancy rate (%)	0	0	0	–
Ongoing pregnancy rate (%)	61.22 (30/49)	75.00 (69/92)	81.25 (13/16)	0.152

To further validate the age risk for blastocyst formation, we divided our participants into two age groups: women with age less than 35 years old and those beyond 35 years old. Surprisingly, we observed a similar result in women under 35 that the blastocyst formation rate is higher in the highest MeDiet appliance women(47.03%), compared to low (40.23%) and moderate (39.74%) appliance women (*p* = 0.033). No difference is observed in pregnancy outcomes (Supplementary Table S2). However, there is no difference in oocyte and embryo quality as well as pregnancy outcomes in women with age above 35 years old (Supplementary Table S3).

## 4 Discussion

In the present study, we noticed that women after COVID-19 infection with high adherence to MeDiet obtained a higher blastocyst formation rate. Meanwhile, MeDiet might play a favorable role in pregnancy outcomes such as clinical pregnancy, implantation as well as ongoing pregnancy. However, there is no relationship between MeDiet and the quantity and grade of oocyte and embryo.

MeDiet is characterized by the incorporation of predominantly plant-based nutritional elements, including fruits, vegetables, legumes, nuts, and olive oil. These items serve as notable reservoirs of bioactive polyphenols. Polyphenols, specifically flavonoids and their derivatives, exhibit diverse health-promoting effects, particularly in cardiovascular and metabolic conditions, owing to their antioxidant, anti-inflammatory, anti-thrombotic attributes, and immunomodulatory effects (31, 32). Accumulating evidence from prospective cohort observational studies suggests that the nutritional status of both the father and mother during the periconceptional period influences early fetal development and the perinatal and long-term health of the offspring (33). Recent studies gradually focus on dietary habits and early embryo development and pregnancy. In an observational study, it was shown that embryos from women reporting higher consumption of fruit and fish had an elevated likelihood of forming a blastocyst. Conversely, there was a decreased probability of blastocyst formation in those who consumed more red meat or were on a weight loss diet (34). Similarly, we found that women with high adherence to Mediet obtained more blastocyst formation in the present study, which is in accordance with the previous results. It is reported that a short MeDiet dietary supplementation alters the rate of embryo cleavage, indicating improved embryo quality (35). However, this study was limited to the cleavage embryos with no data on blastocyst formation.

Mediterranean diet showed a positive correlation with elevated levels of red blood cell folate and vitamin B6 in both blood and follicular fluid. Additionally, following this diet was associated with a reported 40% increase in the likelihood of achieving pregnancy (36). Vitamin B6 serves as a versatile coenzyme engaged in numerous biochemical pathways. Studies indicate that administering vitamin B6 to women experiencing subfertility enhances reproductive performance (37). Additionally, earlier research findings have indicated that the consumption of fruits and vegetables is linked to reduced oxidative stress and enhanced antioxidant status (38), while oxidative stress has been shown to cause defective embryo development *in vitro* (39). Optimal concentrations of antioxidants in oocytes are essential for regular fertilization and subsequent embryonic development during the preimplantation stage (40). It was demonstrated that women with a high intake of alpha-linolenic acid exhibited elevated baseline oestradiol levels, suggesting that increased intake of alpha-linolenic acid and docosahexaenoic acid may enhance embryo morphology (41). This results in accordance with another study which shows the relationship between fish consumption and the likelihood of blastocyst formation (34).

COVID-19 infection could cause a series of body defensive responses, which damages the reproductive process. One of the important mechanisms is associated with exaggerated immune responses like “cytokine storm” and intense inflammation. Excessive production of proinflammatory cytokines can modulate the cellular microenvironment in a way that impairs reproductive physiology (24). Another mechanism is oxidative stress and disturbed reproductive health. Massive reactive oxygen species (ROS) are produced at the subcellular level during the COVID-19 infection period, which is involved in the etiology of errant embryo implantation and development, ovulatory failure, and hyperandrogenism (42, 43). In patients with past COVID-19 infection, decreased pregnancy rates were observed after embryo transfer (44). In another study, a reduced proportion of top-quality embryos was observed (25). Based on those findings, we designed this study to evaluate whether MeDiet improves IVF outcomes in women with past COVID-19 infection. Unfortunately, we did not observe a significant difference in improvement in pregnancy outcomes, which is attributed to the small sample size of women who received embryo transfer. However, there is a trend that the clinical pregnancy rate, implantation rate, and ongoing pregnancy rate are higher along with higher adherence to MeDiet.

Participants in our study adhered to MeDiet at least 1 month before they started IVF treatment. However, it’s a short period. Whether a longer dietary history could prevent adverse effects of infections and/or promote a healthy pregnancy in general should be verified in further research. Moreover, it is a Western dietary pattern, indicating that it is possible that other healthy dietary patterns that suit Asia people could also be beneficial factors in this regard and needs to be determined.

To the best of our knowledge, the present study is the first to propose the influence of the Mediterranean diet on the IVF outcomes of post-COVID-19 females. However, limitations also exist in our study. On one hand, the sample size for women who received embryo transfer is relatively small, especially in women with high MeDiet adherence, which limited the efficacy of the results. On the other hand, a majority of participants in our study with low to moderate adherence to the MeDiet, leaving a minority of them with high adherence. The sample size difference might restrict the effectiveness of the test to some extent. However, the main reason underlying is that the dietary habits differ substantially from Asian to European, thus, only a small group of people could follow the MeDiet. More importantly, we cannot avoid the potential biases associated with self-reported dietary habits. Lastly, we did not follow up on the long-term outcomes such as live birth, or gestational complications. Further studies should give full consideration to sample size and long-term outcomes such as live birth rates and gestational complications.

## 5 Conclusion

High adherence to MeDiet is associated with improved blastocyst formation in women after COVID-19 infection. There is also a trend that high adherence to MeDiet might be beneficial to clinical pregnancy, embryo implantation as well as ongoing pregnancy in these women.

## Data availability statement

The original contributions presented in the study are included in the article/Supplementary material, further inquiries can be directed to the corresponding author.

## Ethics statement

The studies involving humans were approved by Ethics Committee of the Reproductive and Genetic Hospital of CITIC-Xiangya. The studies were conducted in accordance with the local legislation and institutional requirements. The participants provided their written informed consent to participate in this study.

## Author contributions

HC: Conceptualization, Data curation, Formal analysis, Funding acquisition, Investigation, Methodology, Software, Validation, Visualization, Writing – original draft, Writing – review & editing. JW: Methodology, Formal analysis, Validation, Software, Writing – review & editing. HG: Data curation, Project administration, Writing – review & editing. QZ: Data curation, Project administration, Writing – review & editing. GL: Supervision, Writing – review & editing. BH: Writing – review & editing. PK: Writing – review & editing. ZW: Validation, Writing – review & editing. FG: Methodology, Supervision, Validation, Writing – review & editing.
