# Supplementary material for: Mediterranean diet improves blastocyst formation in women previously infected COVID-19: a prospective cohort study
Source: Front Nutr. 2024 Jun 20;11:1371077. doi: 10.3389/fnut.2024.1371077 (PMC11222606; doi:10.3389/fnut.2024.1371077)
Supplement: Supplementary file 1 [file Table_1.DOCX]

**Supplementary materials**

Supplementary table S1. Multivariate regression analysis.

|  | Adjusted β | P value | 95% CI of β | |
| --- | --- | --- | --- | --- |
| Mediet score | 0.077 | 0.019 | 0.028 | 0.313 |
| Age | -0.099 | 0.005 | -0.098 | -0.018 |
| BMI | -0.03 | 0.363 | -0.092 | 0.034 |
| AMH | 0.143 | 0.004 | 0.040 | 0.207 |
| AFC | 0.061 | 0.240 | -0.006 | 0.023 |

BMI: body mass index; AMH: anti-Müllerian hormone; AFC: antral follicle count;

Supplementary table S2. Ovarian stimulation and pregnancy outcomes in women under 35 years old.

|  | **MeDiet score 0-4 (n=129)** | **MeDiet score 5-7 (n=224)** | **MeDiet score 8-14 (n=56)** | **P value** |
| --- | --- | --- | --- | --- |
| Age (year) | 30.00 (28.00, 32.00) | 31.00 (28.00, 32.75) | 32.00 (30.00, 33.00) | 0.061 |
| BMI (kg/m2) | 22.03 (20.10, 24.14) | 22.04 (19.93, 24.14) | 21.57 (20.62, 23.90) | 0.904 |
| AMH (ng/ml) | 3.67 (2.29, 5.59) | 3.22 (1.98, 5.42) | 2.75 (1.58, 5.05) | 0.111 |
| AFC | 26.00 (15.50, 35.00) | 26.00 (16.00, 35.00) | 21.50 (13.00, 36.00) | 0.588 |
| Follicles on hCG day | 12.00 (9.00, 15.00) | 12.00 (8.25, 15.00) | 12.00 (7.00, 14.00) | 0.556 |
| **No. of oocytes retrieved** | 12.00 (9.00, 15.00) | 12.00 (7.00, 16.00) | 11.50 (6.25, 13.75) | 0.435 |
| MII oocytes | 10.00 (7.00, 13.00) | 10.00 (6.00, 13.75) | 9.00 (4.25, 12.00) | 0.493 |
| MI oocytes | 0.84±1.58 | 0.86±1.32 | 0.63±1.26 | 0.271 |
| GV oocytes | 0.92±1.67 | 0.75±1.35 | 0.82±1.92 | 0.777 |
| Degenerated oocytes | 0.12±0.33 | 0.17±0.44 | 0.18±0.39 | 0.621 |
| No. of 2PN zygotes | 7.00 (5.00, 10.00) | 7.00 (4.00, 10.00) | 7.00 (3.00, 10.75) | 0.460 |
| Fertilization rate (%) | 62.50 (50.00, 78.17) | 61.54 (47.37, 76.92) | 68.99 (41.96, 87.50) | 0.257 |
| Cleavage embryos | 9.00 (6.00, 12.00) | 8.00 (5.00, 12.00) | 9.00 (4.00, 12.00) | 0.501 |
| The number of day 3 good quality embryo | 4.00 (1.00, 6.00) | 3.00 (1.00, 6.00) | 3.00 (1.00, 6.75) | 0.684 |
| Blastocyst formation rate (%) | 40.23 (381/947) | 39.74 (639/1608) | 47.03 (174/370) | **0.033** |
| The number of good-quality blastocyst | 0.36±1.00 | 0.39±0.96 | 0.43±1.19 | 0.669 |
| Pregnancy outcomes | **MeDiet score 0-4 (n=37)** | **MeDiet score 5-7 (n=79)** | **MeDiet score 8-14 (n=11)** | **P value** |
| Clinical pregnancy rate (%) | 62.16 (23/37) | 81.01 (64/79) | 72.73 (8/11) | 0.100 |
| Implantation rate (%) | 86.84 (33/38) | 86.27 (88/102) | 70.59 (12/17) | 0.286 |
| Early miscarriage rate (%) | 0 | 1.56 (1/64) | 0 | NA |
| Ectopic pregnancy rate (%) | 0 | 1.56 (1/64) | 0 | NA |
| Ongoing pregnancy rate (%) | 62.16 (23/37) | 79.75 (63/79) | 72.73 (8/11) | 0.140 |

BMI: body mass index; AMH: anti-Müllerian hormone; AFC: antral follicle count; MII: metaphase II (reflects oocytes quality, only MII oocytes can be fertilized); MI: metaphase I; GV: germinal vesicle; 2PN: pronucleus.

Good quality embryo is defined as D3 embryo ≥7C-II and blastocyst ≥4BB while fair embryo is defined as D3 embryo ＜7C-II and blastocyst ＜4BB;

Data are given as medians (interquartile ranges), means ± standard deviation or number (percentage)

Supplementary table S3. Ovarian stimulation and pregnancy outcomes in women over 35 years old.

|  | **MeDiet score 0-4 (n=62)** | **MeDiet score 5-7 (n=102)** | **MeDiet score 8-14 (n=32)** | **P value** |
| --- | --- | --- | --- | --- |
| Age (year) | 37.00 (36.00, 40.00) | 38.00 (36.00, 40.00) | 39.00 (36.00, 40.75) | 0.589 |
| BMI (kg/m2) | 22.64 (20.38, 24.03) | 22.66 (20.96, 24.47) | 22.00 (20.07, 23.44) | 0.266 |
| AMH (ng/ml) | 1.63 (0.86, 4.03) | 1.98 (1.11, 3.37) | 2.51 (1.08, 3.73) | 0.664 |
| AFC | 11.50 (6.00, 23.00) | 15.00 (8.00,25.25) | 12.50 (7.00, 22.75) | 0.304 |
| Follicles on hCG day | 7.00 (4.75, 11.00) | 8.00 (4.00, 13.00) | 12.00 (7.00, 14.00) | 0.465 |
| **No. of oocytes retrieved** | 6.00 (2.00, 12.00) | 8.00 (4.00, 12.00) | 7.50 (3.25, 10.00) | 0.321 |
| MII oocytes | 5.00 (2.00, 10.00) | 7.00 (3.00, 10.00) | 6.50 (2.00, 8.00) | 0.246 |
| MI oocytes | 0.48±1.62 | 0.62±1.17 | 0.38±0.87 | 0.326 |
| GV oocytes | 0.71±1.23 | 0.54±0.94 | 0.66±1.23 | 0.912 |
| Degenerated oocytes | 0.16±0.41 | 0.16±0.44 | 0.13±0.34 | 0.944 |
| No. of 2PN zygotes | 4.00 (1.75, 6.25) | 4.50 (2.00, 8.00) | 4.00 (1.00, 6.00) | 0.220 |
| Fertilization rate (%) | 58.82 (42.86, 75.00) | 63.96 (50.00, 77.14) | 60.00 (46.43, 75.00) | 0.400 |
| Cleavage embryos | 5.00 (2.00, 9.00) | 5.50 (3.00, 9.00) | 5.00 (1.25, 7.00) | 0.326 |
| The number of day 3 good quality embryo | 2.00 (0.75, 5.00) | 2.00 (1.00, 4.25) | 2.00 (1.00, 3.75) | 0.312 |
| Blastocyst formation rate (%) | 46.71 (135/289) | 41.06 (225/548) | 43.57 (61/140) | 0.289 |
| The number of good-quality blastocyst | 0.16±0.55 | 0.17±0.69 | 0.09±0.30 | 0.825 |
| Pregnancy outcomes | **MeDiet score 0-4 (n=12)** | **MeDiet score 5-7 (n=13)** | **MeDiet score 8-14 (n=5)** | **P value** |
| Clinical pregnancy rate (%) | 66.67 (8/12) | 46.15 (6/13) | 100 (5/5) | 0.465 |
| Implantation rate (%) | 71.43 (10/14) | 42.86 (9/21) | 66.67 (6/9) | 0.193 |
| Early miscarriage rate (%) | 8.33 (1/12) | 0 | 0 | NA |
| Ectopic pregnancy rate (%) | 0 | 0 | 0 | NA |
| Ongoing pregnancy rate (%) | 58.33 (7/12) | 46.15 (6/13) | 100 (5/5) | 0.260 |

BMI: body mass index; AMH: anti-Müllerian hormone; AFC: antral follicle count; MII: metaphase II (reflects oocytes quality, only MII oocytes can be fertilized); MI: metaphase I; GV: germinal vesicle; 2PN: pronucleus.

Good quality embryo is defined as D3 embryo ≥7C-II and blastocyst ≥4BB while fair embryo is defined as D3 embryo ＜7C-II and blastocyst ＜4BB;

Data are given as medians (interquartile ranges), means ± standard deviation or number (percentage)
